# Effect of poor glycemic control in cognitive performance in the elderly with type 2 diabetes mellitus: The Mexican Health and Aging Study

**DOI:** 10.1186/s12877-020-01827-x

**Published:** 2020-10-23

**Authors:** Alberto J. Mimenza-Alvarado, Gilberto A. Jiménez-Castillo, Sara G. Yeverino-Castro, Abel J. Barragán-Berlanga, Mario U. Pérez-Zepeda, J. Alberto Ávila-Funes, Sara G. Aguilar-Navarro

**Affiliations:** 1 Geriatric Medicine & Neurology Fellowship, Instituto Nacional de Ciencias Médicas y Nutrición Salvador Zubiran, 14000 Mexico City, Mexico; 2 Department of Geriatric Medicine, Instituto Nacional de Ciencias Médicas y Nutrición Salvador Zubirán, Vasco de Quiroga 15. Tlalpan, 14000 Mexico City, Mexico; 3 Tecnológico de Monterrey, Escuela de Medicina y Ciencias de la Salud, 64160 Monterrey, Nuevo León Mexico; 4 Instituto Nacional de Geriatría, 10200 Mexico City, Mexico; 5 Univ. Bordeaux, Inserm, Bordeaux Population Health Research Center, UMR 1219, F-33000 Bordeaux, France

**Keywords:** Glycemic control, Cognitive performance, Elderly persons, Type 2 diabetes mellitus

## Abstract

**Background:**

Cognitive impairment is twice more frequent in elderly with type 2 diabetes mellitus (DM). This study was conducted to determine the association between glycemic control and cognitive performance among community-dwelling elderly persons in Mexico.

**Methods:**

Cross-sectional study conducted in individuals aged 60 years or elderly participating in the 2012 Mexican Health and Aging Study. Type 2 DM participants were classified in 3 groups according to their glycated hemoglobin levels (Hb_A1c_): < 7% (intensive control), 7–7.9% (standard control) or ≥ 8% (poor control), and cognitive performance: low (CCCE ≤44 points), intermediate (44.1–59.52 points), or high (≥59.53 points). Multinomial logistic regression models were constructed to determine this association.

**Results:**

Two hundred sixteen community-dwelling adults aged 60 and older with type 2 diabetes were selected. Subjects in the low cognitive performance group were older (69.7 ± 6.6 vs 65.86 ± 5.18 years, *p* < .001) and had a lower educational level (2.5 ± 2.6 vs 7.44 ± 4.15 years, *p* < .000) when compared to the high cognitive performance participants. Hb_A1c_ ≥ 8% was associated with having low (Odds Ratio (OR) 3.17, 95% CI 1.17–8.60, *p* = .024), and intermediate (OR 3.23, 95% CI 1.27–8.20, *p* = .014) cognitive performance; this trend was not found for Hb_A1c_ 7.0–7.9% group.

The multinomial regression analysis showed that the presence of Hb_A1c_ ≥ 8% (poor glycemic control) was associated with low (OR 3.17, 95% CI = 1.17–8.60, *p* = .024), and intermediate (OR 3.23, 95% CI = 1.27–8.20, *p* = .014) cognitive performance. After adjusting for confounding variables.

**Conclusions:**

Glycemic control with a Hb_A1c_ ≥ 8% was associated with worse cognitive performance.

## Background

Type 2 Diabetes Mellitus (DM) rates and its complications are increasing faster than expected. Prevalence increased from 4.3 to 9.0% in men and 5.0 to 7.9% in women from 1980 to 2014. By year 2025, diabetes would exceed 700 million cases. Thus, 37.9% of the rising prevalence is due to population growth and ageing [1]. In our country, Mexican National Health and Nutrition Survey (ENSANUT) reported a DM prevalence of 27.4% in the geriatric population [2]. The increasing prevalence of diabetes will lead to a higher number of people with diabetes-related cognitive impairment. For this reason, efforts to control not only type 2 DM, but hypertension, depression and other risk factors are essential for dementia prevention around the world [3, 4].

Numerous reports have shown that patients with DM are at increased risk of Alzheimer’s disease (AD) dementia and vascular dementia [5]. It is currently accepted that worldwide type 2 DM increases 1.5 to 3 times the risk of dementia [6]. The Rotterdam study was one of the first population-based studies to examine the relationship between type 2 DM and cognitive impairment. It reported an increased risk of 1.9 (95% CI: 1.3–2.8) for this population [7]. In Mexico, a longitudinal study which included data from the Mexican Health and Aging Study (MHAS) reported a relative risk for cognitive impairment of 2.08 (95% CI: 1.59–2.73) among type 2 DM in elderly [8].

An optimal glycemic control is needed to prevent or reduce type 2 DM complications such as nephropathy, retinopathy, neuropathy and cognitive disorder. Although, epidemiologic studies have associated poor type 2 DM control with cognitive decline, clinical evidence about glycemic control goals for type 2 DM in elderly is contradictory and lacking. The American Diabetes Association (ADA) 2019 guidelines suggest a reasonable glycated hemoglobin (Hb_A1c_) goal of < 7% for most patients and a less stringent < 8% for those with limited life expectancy or multiple comorbid conditions [9, 10]. The American Geriatric Society (AGS) recommends a Hb_A1c_ goal < 7.5% for healthy population; while for the complex or comorbid and functional-dependent patient the aim should be a Hb_A1c_ of < 8% [11]. According to these guidelines, Hb_A1c_ goals are based on two variables, which must be considered before a decision: cognitive and functional status. Thus, in both guidelines Hb_A1c_ goal recommendation is < 8% for subjects with mild to moderate cognitive impairment [9–11].

ADA 2019 guideline recommendations on a Hb_A1c_ goal below or greater than 8% are lacking on making a clear reference on the risk of adverse effects and cognitive impairment progression. Studies, such as the Memory in Diabetes (MIND) substudy of the Action to Control Cardiovascular Risk Diabetes (ACCORD), which has been one of the most influential when concerning glycemic goals found no difference in cognitive outcomes with either Hb_A1c_ < 6% or 7.0–7.9 [12]. This study could provide different insights towards the appraisal of cognitive impairment through knowledge of type 2 DM, one of its main risk factors. Although, cultural variances could be noted as environmental factors carry a great weight on the disease; glycemic control is a parameter that could be standardized across countries. The aim of the present study was to determine the association between glycemic control and cognitive performance among Mexican rural and urban community-dwelling older adults analyzed in the MHAS round 2012.

## Methods

### Study population

Data was obtained from the MHAS, a large, national representative panel study of older Mexicans (age 50 or older) and their spouses. Briefly, the aim and design of MHAS has to evaluate its participants health and cognitive characteristics. The study started in 2001 and has four follow-ups (2003, 2012, 2015 and 2018). Information from a subsample of subjects who participated in 2012 wave was used for the present study. Data was assessed through performance test, anthropometric measures and blood samples; included HbA_1c_, among others [13, 14]. Additional information can be found at: http://www.mhasweb.org/ [15].

### Sample selection

The 2012-MHAS round included 15,723 subjects (aged 50 and over). For the present study, people aged 60 years or older with complete information from cognitive tests (7469 subjects) and available HbA1c biomarker measurements (946 subjects) were required.

**Fig. 1:**
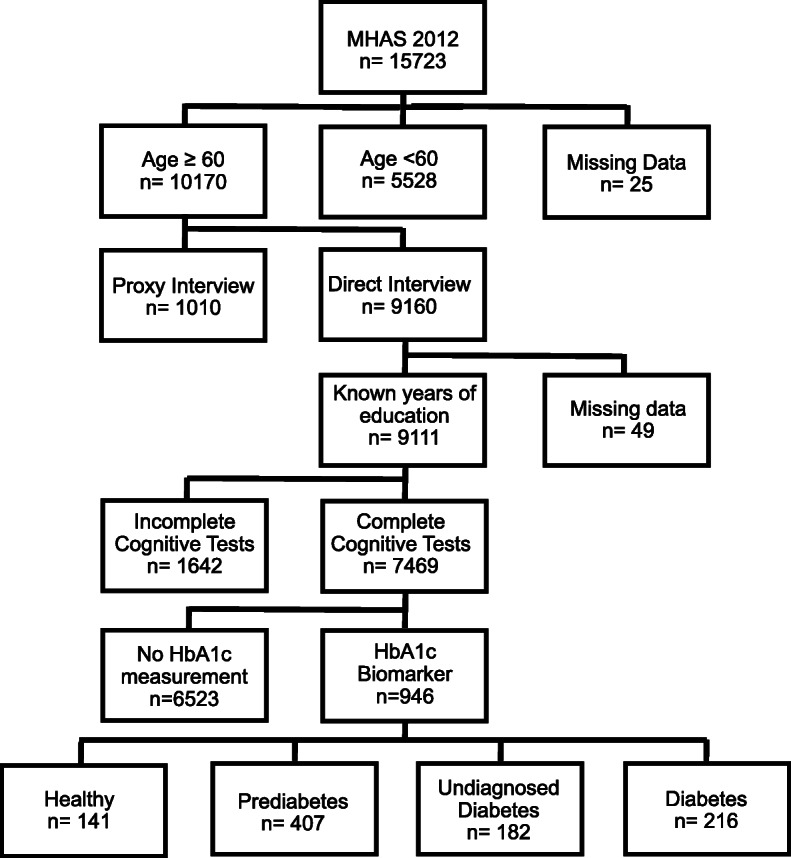
Flowchart of sample selection, MHAS: Mexican Health Aging Study 2012

Type 2 DM was considered when a positive answer was given to the following question: “Has a doctor ever told you or given you a diagnosis of diabetes mellitus?”. Fasting glucose levels and the use of antidiabetic drugs were not considered. According to HbA1c levels: 141 (14.9%) without diabetes (HbA1c: < 5.5%), 407 (43%) with prediabetes (HbA1c: 5.5–6.4)%), 182 (19, 2%) had undiagnosed DM (HbA1c ≥ 6.5%) and only 216 (22.8%) had history of type 2 DM and available HbA1c **(**Fig. 1**)**.

### Glycemic control

Glycemic control was established as an independent variable, data available in the MHAS database section I, it consists of a single measurement of Hb_A1c_ was measured in the MHAS with the A1c-now test kit, an immunoassay device. This method in comparison to the standard liquid chromatography test has a sensitivity of 91.9 to 100%, and a specificity of 66.7 to 82.4% [16].

Of note, pre-prandial capillary glucose was not available for the analysis. Glycemic control categories were defined based on the cut-off points used in the ADA and ACCORD-MIND studies [9, 12]. The following glycemic control groups were defined: intensive (Hb_A1c_ < 7%), standard (Hb_A1c_ 7–7.9%), and poor (Hb_A1c_ ≥ 8%).

Glycemic control categories were defined based on the cut-off points used in the ADA and ACCORD-MIND studies [9, 12]. The following glycemic control groups were defined: intensive (Hb_A1c_ < 7%), standard (Hb_A1c_ 7–7.9%), and poor (Hb_A1c_ ≥ 8%).

### Cognitive performance

In order to determine the cognitive status, all participants underwent the Cross-Cultural Cognitive Examination (CCCE). The CCCE, was selected for both its transcultural attributes (responses are not influenced by education, language or culture) and concurrent validity, which in comparison to other common cognitive instruments, has a 94% specificity and 99% sensitivity for the detection of cognitive impairment [17]. Total CCCE scores consist of a sum of maximum 99 points. For direct respondents, the MHAS assesses cognitive function through a modified version CCCE; which measures performance in eight cognitive domains—verbal learning, delayed memory, attention, constructional praxis, visual memory, verbal fluency, orientation, and processing speed—and has reference norms by age and education [18]. In order to determine the association between cognitive performance level and glycemic control and as a strategy for the abnormal distribution of the data, the total CCCE score was classified by tertiles where the highest represents a better cognitive performance and vice versa. Therefore, cognitive performance was defined as low (CCCE ≤44 points), intermediate (44.1–59.52 points), and high (≥59.53 points). This was the dependent variable for the study.

### Covariables

Age, sex and level of education were the only sociodemographic variables analyzed. Participants’ positive responses to the questions: Has a doctor ever told you that you have ... [i.e. smoking history, alcoholism, hypertension, cerebrovascular disease (CVD), and ischemic heart disease (IHD), were considered as for the subjects’ clinical characteristics. Obesity was considered when the subject’s body mass index (BMI) was ≥30 kg/m^2^ [19]. Further, five cardiovascular comorbidities or categoric variables were grouped as a compound or sum in order to construct a continuous variable. The presence of hypertension, smoking history, CVD, obesity or previous heart disease added one point each to the comorbidity score. A score of 5 represents the highest level of cardiovascular morbidity, while 0 equals no comorbidity.

Depressive symptoms were classified as high for scores ≥5 in a modified version of the *Center for Epidemiologic Studies Depression Scale* (CES-D) validated in Mexico [20]. Blood pressure and heart rate measurements, as well as C-reactive protein, total cholesterol, high density cholesterol (HDL), thyroid stimulating hormone (TSH), and vitamin D levels were also analyzed.

### Statistical analysis

Categorical and continuous variables were analyzed with chi-squared and ANOVA, respectively. Multinomial logistic regression models were used to determine the association between glycemic control and cognitive performance and adjusted by several potential confounders including age, educational level, blood pressure, TSH, C-reactive protein and vitamin D blood levels, depressive symptoms, and the comorbidities score. Statistical significance was considered at a *p* value ≤.05 and 95% confidence intervals (CI) were given. Statistical analysis was performed using SPSS software for Windows® (SPSS Inc., Chicago, IL version 23.0).

## Results

From a total of 216 participants with type 2 DM were included in the analysis, the mean age was 68.11 (± 6.4) years, 57.9% were female, and the mean education level was 4.72 (± 3.9) years. Type 2 DM participants also had a high prevalence of hypertension (69%) and IHD (7.9%). Obesity was reported in 40% of the participants. Smoking history was present in 9.7% and alcohol history in 18.1%. The mean score for depressive symptoms was 4.76 (± 1.9) points. Mean Hb_A1c_ blood level value was 8.34 ± 2.05%. When divided by glycemic control groups, no differences were found in any of the variables analyzed (Table 1).

**Table 1:** Sociodemographic, health status and glycemic control of the study sample. MHAS 2012

	Total	Intensive Glycemic Control Hb_A1c_ < 7.0%	Standard Glycemic Control Hb_A1c_ 7.0–7.9%	Poor Glycemic Control Hb_A1c_ ≥ 8%	*P*
Mean (SD)/nn/SD(%)	*n* = 216	*n* = 67	*n* = 46	*n* = 103	
Age	68.11 (6.48)	68.82 (6.92)	68.02 (6.96)	67.69 (5.98)	.538
Female	125 (57.9%)	37 (52.22%)	23 (50.0%)	65 (63.1%)	.284
Education level	4.72 (3.96)	4.72 (3.81)	4.74 (3.51)	4.72 (4.28)	.999
Smoking history	21 (9.7%)	7 (10.5%)	5 (10.9%)	9 (8.7%)	.895
Alcoholism	39 (18.1%)	13 (19.4%)	9 (19.6%)	17 (16.5%)	.852
Hypertension	149 (69.0%)	44 (65.7%)	30 (65.2%)	75 (72.8%)	.508
CVD	4 (1.9%)	1 (1.5%)	1 (2.2%)	2 (1.9%)	.962
Obesity	87 (40.3%)	30 (44.8%)	17 (37.0%)	40 (38.8%)	.649
Depressive Symptoms	4.76 (1.97)	4.64 (2.32)	4.61 (1.78)	4.91 (1.79)	.569
IHD	17 (7.9%)	7 (10.4%)	4 (8.7%)	6 (5.8%)	.535

Data are presented as means or percentage, ANOVA analysis was performed. *CVD* Cerebrovascular Disease, *IHD* Isquemic Heart Disease, *SD* Standard Deviation

As shown in Table 2, were classified as having low (35.1%), intermediate (31.4%) or high (33.3%) cognitive dysfunctions. Subjects in the lower cognitive performance group were older (69.7 ± 6.6 years vs 65.86 ± 5.18 *p* < .001) and had a lower educational level (2.5 ± 2.6 years vs 7.44 ± 4.15; *p* < .000) when compared to the high cognitive performance participants. No significant differences between the three groups were found for any of the other clinical variables, except for alcoholism, which was more frequent in the intermediate cognitive performance group (27.9%, *p* = .023).

**Table 2:** Cognitive Performance, clinical characteristics and glycemic control data of the study sample. MHAS 2012

Cognitive Performance	Total	Low ≤44.0	Intermediate 44.1–59.52	High ≥59.53	*P*
Mean (SD)/nn/SD (%)	*n* = 216	*n* = 76	*n* = 68	*n* = 72	
Age	68.11 (6.48)	69.75 (6.66)^a^	68.66 (6.93)	65.86 (5.18)	.001*
Female	125 (57.9%)	49 (64.5%)	36 (52.9%)	40 (55.6%)	.337
Education level	4.72 (3.96)	2.57 (2.67)^ab^	4.25 (3.27)	7.44 (4.15)	.000*
Smoking history	21 (9.7%)	7 (9.2%)	6 (8.8%)	8 (11.1%)	.887
Alcoholism	39 (18.1%)	8 (10.5%)^a^	19 (27.9%)	12 (16.7%)	.023*
Hypertension	149 (69.0%)	54 (71.1%)	49 (72.1%)	46 (63.9%)	.519
CVD	4 (1.9%)	3 (3.9%)	1 (1.5%)	0 (0.0%)	.199
Obesity	87 (40.3%)	27 (35.5%)	28 (41.2%)	32 (44.4%)	.537
Depressive Symptoms	4.76 (1.97)	5.04 (1.91)	4.81 (2.08)	4.43 (1.89)	.166
IHD	17 (7.9%)	5 (6.6%)	7 (10.3%)	5 (6.9%)	.670
Systolic Pressure	144.67 (21.72)	145.54 (22.83)	147.68 (21.83)	140.93 (20.10)	.169
Diastolic Pressure	77.67 (12.20)	76.68 (12.97)	79.75 (12.46)	76.75 (10.96)	.237
Heart Rate	76.71 (12.16)	76.28 (12.67)	78.90 (12.75)	75.10 (10.85)	.169
PCR	4.68 (7.20)	4.78 (7.28)	4.98 (8.54)	4.30 (5.68)	.854
Total Cholesterol	192.64 (41.88)	194.16 (37.94)	196.46 (55.01)	187.50 (29.13)	.431
HDL	38.38 (8.81)	36.86 (8.12)	38.60 (9.28)	39.74 (8.93)	.144
TSH	3.35 (8.38)	3.64 (11.59)	4.14 (8.35)	2.31 (1.95)	.416
Vitamin D	21.13 (6.83)	20.85 (7.40)	20.94 (6.40)	21.60 (6.67)	.779
Glycemic Control					
Poor Hb_A1c_ ≥ 8%	103 (47.7%)	41 (54.0%)	36 (52.9%)	26 (36.1%)	.166
Standard Hb_A1c_ 7.0–7.9%	46 (21.3%)	13 (17.1%)	15 (22.1%)	18 (25%)	
Intensive Hb_A1c_ < 7.0%	67 (31.0%)	22 (28.9%)	17 (25%)	28 (38.9%)	

Data are presented as means or percentage, ANOVA analysis was performed. *CVD* Cerebrovascular Disease, *IHD* Isquemic Heart Disease, *PCR* Reactive protein-C, *HDL* High density cholesterol, *TSH* Stimulating thyroid hormone, *Hb*_*A1c*_ Glycated hemoglobin, *SD* Standard Deviation. *p ≤ 0.05. Post-hoc DSM ^a^*p* < 0.001 Low vs High cognitive performance; ^b^*p* < 0.001 Low vs Intermediate cognitive performance

When compared to the intensive glycemic control group those with poor glycemic control had a borderline association with worse cognitive performance in the unadjusted regression model. After adjusting for confounding variables, the multinomial regression analysis showed that the presence of Hb_A1c_ ≥ 8% (poor glycemic control) was associated with low (Odds Ratio (OR) 3.17, 95% CI = 1.17–8.60, *p* = .024), and intermediate (OR 3.23, 95% CI = 1.27–8.20, *p* = .014) cognitive performance. This trend was not found for the standard glycemic control (Hb_A1c_ 7–7.9%) (Table 3).

**Table 3:** Logistic regression analysis in comparison with the reference group

	Low Cognitive Performance			Intermediate Cognitive Performance		
Unadjusted Model	Odds Ratio	95% CI	*p*	Odds Ratio	95% CI	*p*
Poor Control Group (Hb_A1c_ > 8%)	2.01	0.95–4.22	.066	2.28	1.04–5.00	.040*
Standard Control Group (Hb_A1c_ 7–7.9%)	0.92	0.37–2.27	.812	1.37	0.55–3.42	.496
Adjusted Model	Odds Ratio	95% CI	*p*	Odds Ratio	95% CI	*p*
Poor Control Group (Hb_A1c_ > 8%)	3.17	1.17–8.60	.024*	3.23	1.27–8.20	.014*
Standard Control Group (Hb_A1c_ 7–7.9%)	1.15	0.37–3.62	.812	1.57	0.60–4.41	.391

High cognitive performance and intensive glycemic control were considered as the reference groups. Hb_A1c_: Glycated Hemoglobin. Confounding variables: age, educational level. CI: Confidence Interval. *p ≤ 0.05

## Discussion

In our study of community-dwelling older Mexican adults with type 2 DM, a poor glycemic control (Hb_A1c_ ≥ 8%) was associated with worse cognitive performance when compared to intensive control group. The uncontrolled type 2 DM group had a positive association with overall low cognitive performance, while the standard controlled population (Hb_A1c_ 7–7.9%) did not show an association.

A high Hb_A1c_ level (> 10%) is associated with an increased risk of all type dementia (HR 1.20, 95% CI 1.07–1.35) [21]. However, studies that analyze glycemic control, specifically, Hb_A1c_ levels ≥8% and their association with cognitive performance, are scarce. After a sub-analysis, a US prospective study of 5099 participants showed an association between Hb_A1c_ levels (≥8% and 7–7.9%) and mild cognitive impairment (MCI) measured by proxy (Hazzard Risk (HR) 1.89, CI 95% 1.14–3.14, *p* < 0.05 and HR 1.65, CI 95% 1.13–2.42, *p* < 0.01, respectively) in an older adult population [22]. Our results support these association, as an Hb_A1c_ ≥ 8% was associated with worse cognitive performance.

Multiple studies have identified that an intensive vs a standard glycemic treatment had no beneficial or detrimental effects on cognition [12, 23, 24]. A meta-analysis involving five studies, that included 24,297 participants, found that neither intensive (Hb_A1c_ 6.0–7.0%) nor standard (Hb_A1c_ 7.1–8.0%) glycemic control, when compared to each other, had significant cognitive decline rates (SMD = 0.02; 95% CI = − 0.03 to 0.08) [25]. One of the studies mentioned above included, where guideline recommendations are based mostly is the ACCORD-MIND study. Two thousand nine hundred seventy-seven participants (aged 55–80 years) with higher Hb_A1c_ levels (> 7.5%) were randomly assigned to an intensive treatment goal (Hb_A1c_ < 6.0%) or a standard strategy (Hb_A1c_: 7.0–7.9). In this North American trial, as previously, authors found no difference when comparing cognitive outcomes between groups after 40-week treatment, establishing a greater amplitude of therapy goal for patients [12]. In our study, we were not able to find an association between standard glycemic control (Hb_A1c_ 7–7.9%) and low cognitive performance, supporting the previously reported analysis.

Several studies have evaluated the impact of type 2 DM on cognition; however, methodological differences are noted [25]. Data from the English Longitudinal Study of Ageing (ELSA) showed in 5189 participants, a longitudinal association between Hb_A1c_ levels and a rate of change in cognitive scores, where 1 mmol/mol increment in Hb_A1c_ was significantly associated with increased rate of decline in global cognitive z scores (− 0.0009 SD/ year, 95% CI -0.0014, − 0.003, *p* 0.002) [26]. Our study provides an association of glycemic control levels and global cognitive performance supporting the data mentioned above; unlike other studies, additional specific Hb_A1c_ goals are analyzed and type 2 DM is not taken as a single entity.

After several studies of diabetes and cognitive impairment, authors have gone as far as proposing the possibility of type 3 of DM. Chronic hyperglycemia contributes to conditions such as inflammation, accumulation of advanced glycation end products, and oxidative stress, which in turn lead to cognitive impairment [27]. Studies have shown that persons with DM experience a progressive cognitive decline, particularly characterized by a lower psychomotor speed and alterations in cognitive domains such as attention and executive function [28]. Disruption in glucose metabolism leads to lower cognitive dysfunctions through different mechanisms; a) GLUT transporter altered sensitivity, b) insulin resistance, and c) vascular dysfunction. Chronic hyperglycemia is a phenomenon that inhibits brain autoregulation since GLUT transporters diminish their function in order to protect neurons from an increased glucose influx. When glucose is restored to a normal level, GLUT transporters fail to recover, causing an absence of intraneuronal glucose in a process called neuroglycopenia [29]. Insulin resistance could lead to apoptosis by disruption of a secondary pathway; insulin receptor phosphorylation which disrupts long term potentiation, thus increasing inflammation and generating oxidative stress [30]. Vascular homeostasis is also affected by type 2 DM. The presence of atherosclerotic plaques, endothelial dysfunction, increased shear stress, inflammation, impaired vasodilation, and increased vasoconstriction, are some of the mechanisms that lead to vascular injury. The theories presented above, often converge into a type 3 DM diagnosis [31, 32].

The combination of factors seen in the type 2 DM population (hypertense, obese, IHD) mirrors the population’s clinical characteristics that physicians are set to treat in the present and near future. As type 2 DM control is one of the strongest modifiable comorbidities that affect brain function, hypertension and obesity are equally relevant risk factors to target. Since currently there are no therapies to cure dementia, the treatment of modifiable risk factors should be emphasized [2, 3].

Some other factors may impact the glycemic control in older adults. In our study, type 2 DM participants with low cognitive performance were older and had a lower educational level. Studies have shown that age is the most important and non-reversible risk factor for the development of cognitive dysfunctions [33]. In elderly adults, pharmacologic management with multiple drugs leads to a low treatment adherence, given a higher number of side effects. Besides, non-pharmacologic treatments such as a diet and lifestyle interventions are generally less effective, since the modification of eating and physical activity habits is usually a difficult task [34, 35]. Education has previously been described as a protective factor for cognitive impairment, as higher education allows the development of “cognitive reserve” and a lower educational level is associated with a 5.6 greater risk of dementia [36]. Also, a lower educational level could influence glycemic control. Adherence to treatment and lifestyle recommendations, disease complications, and awareness are some of the variables in which a lower educational level, over time, has a negative impact [37, 38].

Our study has several limitations. The cross-sectional nature of this study is a major limitation for making cause-effect statements. Also, since the MHAS data was gathered through a survey, many subjects were excluded because there was a lack of biomarker availability. In addition, only a small sample of the cohort was analyzed as we sought to study fulfill the diagnosis of diabetes in community dwelling residents; this could lead to low statistical power and limiting the external validity of the results. Regarding type 2 DM, diagnosis was limited to one Hb_A1c_ measurement. A history of duration in years and information on previous control strategies was not obtained. Nevertheless, our study has several strengths. The MHAS is a large representative sample of community older adults; considering that control of cognitive impairment risk factors is a primary prevention strategy that should be prioritized.

## Conclusions

Our study shows a Hb_A1c_ value ≥8% in older adults with diabetes is associated with a worse cognitive performance. Adequate glucose control should also be promoted in older adults.

## Data Availability

The datasets used and/or analyzed during the current study are available from the corresponding author on reasonable request.

## Abbreviations

ACCORD-MIND: The Memory in Diabetes (*MIND*) sub study of the Action to Control Cardiovascular Risk in Diabetes (*ACCORD*)
ADA: American Diabetes Association
CCCE: Cross-Cultural Cognitive Examination
CVD: Cerebrovascular disease
DM: Diabetes Mellitus
ELSA: English Longitudinal Study of Ageing
GLUT: Glucose *transporter*
Hb_A1c_: Glycated Hemoglobin
HDL: High Density Cholesterol
IHD: Ischemic Heart Disease
MCI: Mild Cognitive Impairment
MHAS: Mexican Health and Aging Study
TSH: Thyroid stimulating hormone
